# A Study on the Peak‐to‐Valley Characteristics of Intellectual Structure in Low‐Functioning Children With Autism Spectrum Disorder

**DOI:** 10.1002/pdi3.70041

**Published:** 2026-03-26

**Authors:** Danyang Zhang, Qiuhong Wei, Binyue Hu, Dan Ai, Yu Zhang, Xueli Xiang, Ting Yang, Qian Zhang, Qian Chen, Min Guo, Jie Chen, Tingyu Li, Hua Wei

**Affiliations:** ^1^ Children Nutrition Research Center, Chongqing Key Laboratory of Child Neurodevelopment and Cognitive Disorders, Ministry of Education Key Laboratory of Child Development and Disorders, National Clinical Research Center for Child Health and Disorders Children's Hospital of Chongqing Medical University Chongqing China; ^2^ Department of Primary Child Health Care Children's Hospital of Chongqing Medical University Chongqing China

**Keywords:** autism spectrum disorder, high functioning, intellectual structures, low functioning, peak‐to‐valley characteristics

## Abstract

Autism Spectrum Disorder (ASD) is marked by a distinctive cognitive profile reflecting neurodiversity, yet whether this profile extends consistently across the functional spectrum remains uncertain. In this study, we examined the intellectual structure of children with low‐functioning ASD (LF‐ASD) and high‐functioning ASD (HF‐ASD), comparing their cognitive peak‐valley profiles using the Chinese Wechsler Intelligence Scale for Children (C‐WISC). Among 314 children aged 6–13 years—including 104 with LF‐ASD, 122 with HF‐ASD, and control groups with typical development (TD) or intellectual disabilities (ID)—both ASD subgroups displayed significantly greater discrepancies between verbal and performance IQ than controls, with pronounced strengths in visuospatial tasks and weaknesses in arithmetic reasoning. Notably, the Block Design and Object Assembly subtests emerged as cognitive peaks across both ASD groups, whereas the Arithmetic subtest constituted the most frequent trough. Although the mean peak‐valley discrepancy was slightly reduced in LF‐ASD compared to HF‐ASD, both exceeded the 2 standard deviation (SD) threshold for neurodiversity, distinguishing them from the TD and ID groups. These profiles were positively associated with adaptive functioning and inversely related to ASD symptom severity. Our findings suggest that children with LF‐ASD exhibit intellectual asymmetries comparable to those of HF‐ASD, supporting the universality of neurodiversity within the autism spectrum and offering valuable insights for tailoring cognitive interventions.

## Graphical abstract

This article first investigates the quantitative peak‐valley pattern comparisons between the intellectual structures of children with low‐functioning ASD (LF‐ASD) and those with high‐functioning ASD (HF‐ASD), and found LF‐ASD and HF‐ASD exhibited comparable imbalances in their intellectual frameworks.

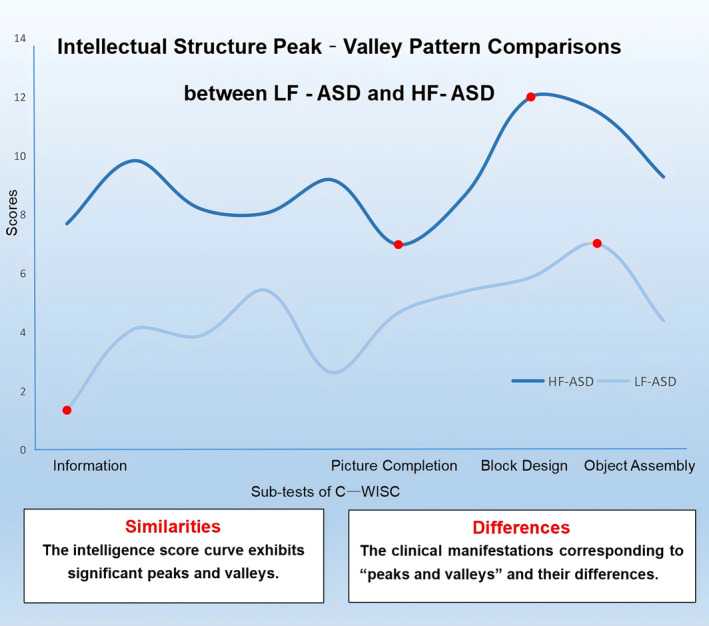

## 1 Introduction

Autism spectrum disorders (ASD) are characterized by two core symptoms: impaired social and communication abilities, and restricted interests accompanied by repetitive behaviors. This early‐onset, lifelong neurodevelopmental disorder significantly affects both the physical and mental health of children [1]. In recent years, the prevalence of ASD has continued to rise. The latest prevalence rate among children in the United States is approximately 2.8% [2], however in China, it stands at about 1.8% [3]. Furthermore, the male‐to‐female ratio is approximately 4:1 [4]. Clinically, a full scale intelligence quotient (FIQ) greater than 70 is referred to as high‐functioning ASD (HF‐ASD), whereas an FIQ less than 70 is classified as low‐functioning ASD (LF‐ASD) [5]. Data from the 2023 Autism and Developmental Disabilities Monitoring (ADDM) Network in the United States indicate that the prevalence of comorbid intellectual disability among 8‐year‐old children with ASD is 37.9% [2]. Although there are significant discrepancies in statistical data from different sources, a report by The Lancet indicates that 11%–65% of school‐aged children with ASD have intellectual disabilities [6, 7]. The Wechsler Intelligence Scale for Children (WISC) is currently the most widely used intelligence testing scale for assessing the cognitive characteristics of children, and its subtests have a certain sensitivity to the cognitive traits of children with ASD [8, 9, 10]. Numerous investigations that examine the cognitive abilities of people with ASD suggest that although intelligence quotient (IQ) tests are not used as a means of diagnosing ASD, they are useful for distinguishing between those who have high functioning and those with low functioning [11].

Neurodiversity refers to statistically significant deviations in cognitive score peaks and valleys among minority atypical development groups, resulting in a ‘sharp structure’ characterized by imbalanced intellectual profiles. In contrast, the cognitive score peaks and valleys of typically developing (TD) groups fall within one or two standard deviations of the mean, forming a flat structure. ASD, as a prototype of neurodiversity, exhibits an imbalanced intellectual structure in cognitive psychology [12, 13], characterized by distinct strengths and weaknesses, manifesting as a ‘sharp structure’ where the difference between peaks and valleys exceeds 2 standard deviations. Research has shown that patients with ASD who undergo intelligence analysis using the WISC often demonstrate an imbalance in the development of verbal intelligence quotient (VIQ) and performance intelligence quotient (PIQ). This developmental imbalance may assist in differentiating ASD from other neurodevelopmental disorders [11, 14, 15, 16]. However, some studies have reported no differences between VIQ and PIQ [17], with even higher scores observed on verbal scales [18]. Most studies, while showing differences in results regarding the comparison of scales, exhibit two common findings: the highest scores are obtained in the “block design” test reflecting visual‐spatial memory advantages, whereas the lowest scores are observed in the “comprehension” aspect (the “valley”) [19, 20, 21]. Currently, most research primarily focuses on children with HF‐ASD, and there is a lack of reports on the cognitive structure of LF‐ASD compared to HF‐ASD, as well as the quantification of the peak‐valley difference between the two. Therefore, this research examines the cognitive structure features of children with LF‐ASD and HF‐ASD, and for the first time measures the difference in intelligence scale peaks and valleys for children diagnosed with ASD. This analysis lays the groundwork for investigating the neurodiversity traits of children with ASD, enabling differential diagnosis and tailored interventions.

## 2 Materials and Methods

### 2.1 Participants

**FIGURE 1:**
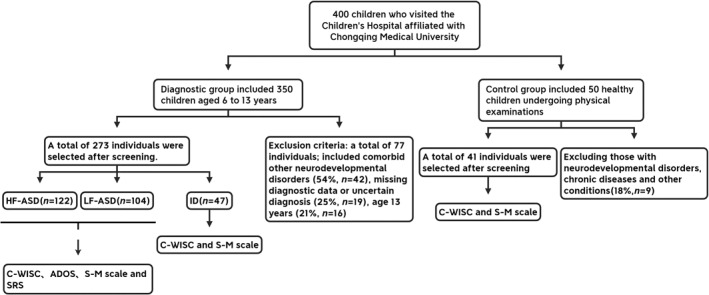
Schematic diagram of inclusion and exclusion processes. HF‐ASD, high‐functioning autism spectrum disorder; ID, intellectual disabilities; LF‐ASD, low‐functioning autism spectrum disorder; TD, typical development.

This study is a single‐center retrospective analysis conducted at the Children's Hospital Affiliated with Chongqing Medical University from 2023 to 2024. Initially, 350 children diagnosed with ASD and ID were included from the Pediatric Health Care Outpatient Department of the Children's Hospital affiliated with Chongqing Medical University, and 50 typically developing (TD) children were included from the Health Examination Department. The enrolled children underwent a standardized diagnostic assessment during the diagnostic process, followed by further screening based on inclusion and exclusion criteria. Ultimately, a total of 314 children aged 6–13 years were included, comprising the HF‐ASD group (*n* = 122), LF‐ASD group (*n* = 104), TD group (*n* = 41), and ID group (*n* = 47). This study has been approved by the Ethics Committee of the Children's Hospital Affiliated with Chongqing Medical University (2024305), and the requirement for informed consent has been waived. The enrollment flowchart is presented in Figure 1.

#### 2.1.1 Inclusion Criteria

Participants fulfilling the following criteria were eligible to be included:

1. The diagnoses of ASD and ID aligned with the diagnostic criteria specified in the “Diagnostic and Statistical Manual of Mental Disorders, Fifth Edition” (DSM‐5) and were confirmed by one or two developmental‐behavioral specialists holding at least an associate professor rank;
2. The participants aged between 6 and 13 years at the time of enrollment.
3. The auxiliary diagnostic scale data for ASD included the Chinese WISC (C‐WISC), Infant‐Junior High School Student Social Life Ability Scale/Social Maturity Scale (S‐M), Social Responsiveness Scale (SRS), and Autism Diagnostic Observation Schedule (ADOS) assessments.
4. Control group: To contrast with the high‐functioning intellectual structure, the TD group consisted of healthy children undergoing physical examinations and having results from the C‐WISC and S‐M tests, excluding those with neurodevelopmental disorders or chronic illnesses. To compare with the LF‐ASD intellectual structure, children with ID who did not have comorbid conditions were recruited, and they also had to have results from the C‐WISC and S‐M tests.

#### 2.1.2 Exclusion Criteria

Participants with comorbid neurodevelopmental disorders such as attention deficit hyperactivity disorder (ADHD) and learning disabilities, incomplete diagnostic information, uncertain diagnoses, and aged outside the 6–13‐year range were excluded.

### 2.2 C‐WISC

This study employs C‐WISC, a version of WISC revised by Hunan Medical University [22] which is designed for children aged 6–16 years. The scale is adapted to the Chinese context, and includes three composite indices: VIQ, PIQ and FIQ, with a standard deviation of 15. It comprises 11 subtests: Information, Classification, Arithmetic, Vocabulary, Comprehension (which is not measured in this study), Digit Span, Picture Completion, Picture Arrangement, Block Design, Object Assembly, and Coding (the standard deviation of the differences is 3). Analyzing the subtest scores can yield more detailed insights into the strengths and weaknesses of cognitive functions.

### 2.3 ADOS

The ADOS is a standardized assessment tool utilized for the diagnosis and differential diagnosis of ASD. It primarily evaluates various facets of an individual's social interaction, communication skills, stereotyped behaviors, interests, and imaginative capabilities. Different modules are tailored to individuals of varying ages and ability levels, and the diagnostic cut‐off scores for autism and ASD vary across these modules [23, 24].

### 2.4 SRS

This scale is applicable for screening and assessing ASD in individuals aged 4 years and older, providing a comprehensive evaluation of social functioning through quantified scoring [25, 26]. A score of ≥ 65 indicates a positive result, with higher scores reflecting greater impairment in social functioning [27].

### 2.5 S‐M

This assessment scale for adaptive behaviors suitable for infants to middle school students is designed as a questionnaire filled out by parents. If the evaluation score (standard score) ≤ 9, it indicates low ability, which may suggest that their adaptive ability could be suspiciously abnormal or abnormal [28].

### 2.6 Statistical Analysis

The analysis of data was performed with SPSS version 25.0. For quantitative datasets that followed a normal distribution, results were presented as mean ± standard deviation (*x̅* ± *s*), and one‐way ANOVA was utilized for comparing multiple groups. In cases where the quantitative data did not meet the criteria for normal distribution, the median and the full range were reported, non‐parametric tests were applied to assess the differences between groups concerning continuous variables across various factors, and results were corrected (such as Bonferroni correction). A *p*‐value of under 0.05 was considered to indicate statistical significance.

## 3 Results

### 3.1 Comparison of Demographics and Scores of Various Scales

Firstly, sex and age distributions did not differ significantly between the two groups of children with ASD (*p =* 0.738, *p =* 0.506, respectively) (Tables A1 and A2). There were however significant differences between HF‐ASD and LF‐ASD in the composite indices of the C‐WISC, including FIQ, VIQ, and PIQ (*p <* 0.001 for all) (Table 1). Both groups exhibited significantly higher PIQ than VIQ (*p <* 0.001 and *p <* 0.001, respectively; details can be found in Table A3). Furthermore, HF‐ASD and LF‐ASD demonstrated significant differences in S‐M (*p <* 0.001), SRS (*p *< 0.001), and ADOS (*p *< 0.001) (Table 1). Additionally, the core symptoms and functional impairments (S‐M, SRS, ADOS) in LF‐ASD were significantly more severe than those in HF‐ASD (*p <* 0.001 for all) (Table A4).

**TABLE 1:** Analysis of demographic samples and scale scores among HF‐ASD, LF‐ASD, ID, and TD groups.

Variables	Group				*p*
	HF‐ASD (*n* = 122)	TD (*n* = 41)	LF‐ASD (*n* = 104)	ID (*n* = 47)	
Gender, *n* (%)					
Female	22 (18.03%)	15 (36.59%)	17 (16.35%)	14 (29.79%)	0.020
Male	100 (81.97%)	26 (63.41%)	87 (83.65%)	33 (70.21%)	
Age (years)	10.72 ± 14.83	7.99 ± 1.45	8.51 ± 1.88	8.76 ± 1.96	0.133
C‐WISC					
FIQ	93.43 ± 15.66	90.85 ± 10.46	56.51 ± 7.52	50.32 ± 9.45	< 0.001
VIQ	90.93 ± 18.49	94.05 ± 10.51	57.13 ± 8.27	56.15 ± 8.94	< 0.001
PIQ	97.75 ± 13.16	89.12 ± 10.53	65.04 ± 9.60	53.89 ± 11.28	< 0.001
S‐M	9.60 ± 0.80	9.80 ± 0.79	8.01 ± 1.12	7.66 ± 0.99	< 0.001
SRS	69.15 ± 19.74	/	84.35 ± 22.16	/	< 0.001
ADOS total score	10.39 ± 4.75	/	14.84 ± 4.36	/	< 0.001

*Note*: Data are presented as mean ± standard deviation (SD) or *n* (%).

Abbreviations: ADOS, autism diagnostic observation schedule; HF‐ASD, high‐functioning autism spectrum disorder; ID, intellectual disabilities; LF‐ASD, low‐functioning autism spectrum disorder; S‐M, infant‐junior high school student social life ability scale/social maturity scale; SRS, social responsiveness scale; TD, typical development.

### 3.2 Comparison of HF‐ASD, LF‐ASD, ID and TD Group Scores on the C‐WISC Scale and Peak‐Valley Differences

**FIGURE 2:**
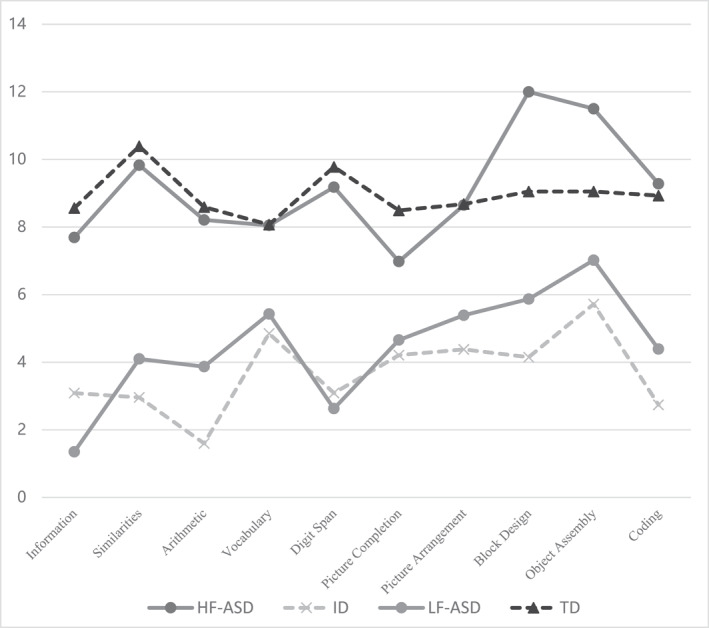
Comparative analysis of scale scores for various subtests of HF‐ASD, LF‐ASD, ID and TD group. HF‐ASD, high‐functioning autism spectrum disorder; ID, intellectual disabilities; LF‐ASD, low‐functioning autism spectrum disorder; TD, typical development.

The mean scores of the scales were visualized and presented in a line chart (Figure 2). The results indicate that the scores of the control group (TD and ID) fluctuated relatively steadily, remaining within a range of 2SD (Table A5). In contrast, the LF‐ASD group exhibited a greater fluctuation in scores, with a peak‐to‐valley difference of up to 2.65 SD (Table A5). Although this group displays characteristics of neurodiversity, its overall waveform resembles that of the ID group more closely. The scores of the HF‐ASD group were generally situated near the TD group's line, but exhibited a notable peak‐to‐valley difference of 3.09 SD (Table A5). Between the HF‐ASD and LF‐ASD groups, the item with the highest peak distribution was the Block Design (43%) and Object Assembly (41%) reflecting visual advantages, with slight differences; however, the valley distributions revealed significant discrepancies. For the HF‐ASD group, the item with the valley distribution was the Picture Completion task (41%), which aligns with the characteristic of ASD that lacks a global perspective. In contrast, the LF‐ASD group primarily exhibited through distributions in Arithmetic tasks (66%), consistent with the prominent deficits observed in the ID group (Table 2).

**TABLE 2:** The top three proportions of peak and valley items for HF‐ASD and LF‐ASD.

	The top three categories in terms of the proportion of HF‐ASD and LF‐ASD	HF‐ASD (*n,* [%])	LF‐ASD (*n*, [%])
Peak	Block design	52 (43%)	33 (32%)
	Object assembly	36 (30%)	43 (41%)
	Similarities	24 (20%)	14 (14%)
Valley	Picture completion	50 (41%)	4 (4%)
	Arithmetic	34 (28%)	69 (66%)
	Information	26 (21%)	13 (13%)

*Note:* Due to the high and low scores of the scale being able to coexist across different subtests, the total proportions may exceed 100.

Abbreviations: HF‐ASD, high‐functioning autism spectrum disorder; LF‐ASD, low‐functioning autism spectrum disorder.

Additionally, both the HF‐ASD and LF‐ASD groups showed significant differences in scores across all subtests (*p <* 0.001) (Table A4). Notably, the HF‐ASD group scored significantly higher on the block design and figure assembly scales compared to other tests, whereas the LF‐ASD group demonstrated significantly higher scores on figure assembly relative to other tests.

### 3.3 The Correlation Between the Scores of Various Subtests of the C‐WISC and Core Symptoms

In children with ASD, the scores of the WISC subtests exhibited a positive correlation with the S‐M scale and a negative correlation with the ADOS and SRS scales. Notably, the HF‐ASD group demonstrated a stronger correlation with the ADOS scale scores in the areas of Information (*p =* 0.001, *r* = −0.402) and Object Assembly (*p <* 0.001, *r* = −0.533) compared to the LF‐ASD group (*p =* 0.659, *r* = −0.104; *p =* 0.117, *r* = −0.175). Conversely, the LF‐ASD group shows a stronger correlation with the SRS scale scores in the areas of Digit Span (*p =* 0.001, *r* = −0.436) and Coding (*p =* 0.002, *r* = −0.431) compared to the HF‐ASD group (*p =* 0.302, *r* = −0.197; *p =* 0.390, *r* = −0.070) (details can be found in Table A6).t

## 4 Discussion

Understanding the advantages and disadvantages of the imbalanced intellectual structure in ASD contributes to recognizing neurodiversity and leveraging the strengths of ASD for societal benefit. Previous studies have reported cognitive strengths and weaknesses in children with high‐functioning ASD [16, 29], highlighting their uneven intellectual structural characteristics and lower language proficiency, which aids in differentiating them from other neurodevelopmental disorders [11, 20]. This study, using the C‐WISC alongside multidimensional diagnostic data from the ADOS, SRS and S‐M scales, reveals that children with LF‐ASD exhibit neurodiversity similar to that of HF‐ASD, providing new strategies for understanding strengths and interventions for LF‐ASD.

This study found that the VIQ of children with ASD, including both high‐functioning and low‐functioning individuals, is significantly lower than their PIQ, with statistical significance. Moreover, the gap was more pronounced in LF‐ASD children due to their more severely impaired language abilities. This finding aligns with the views of Mouga et al. [11], indicating that comorbid intellectual disability further impairs the language abilities of children with ASD. The higher performance scores may be related to peak performance on tasks that leverage the visual strengths associated with ASD, such as Block Design and Object Assembly tests [30, 31, 32]. This suggests that interventions for LF‐ASD require a more comprehensive enhancement of brain function.

The results of this study indicate that the mean peak‐to‐valley difference score for children with HF‐ASD reaches as high as 3.09 SD, whereas the peak‐to‐valley difference score for children with LF‐ASD is 2.65 SD. Although the latter is slightly lower than the former, both scores are significantly higher than those of non‐ASD children, who score below 2 SD. This finding further confirms that children with LF‐ASD, similar to those with HF‐ASD, exhibit an imbalance in the distribution of cognitive structures, which may reflect specific cognitive strengths and weaknesses associated with varying levels of intellectual disability. Reduced functionality in individuals with LF‐ASD may stem from global cognitive deficits masking neurodiverse phenotypes, thereby attenuating observable disparities [11].

Furthermore, an analysis of the subtests of the C‐WISC reveals that the unique cognitive characteristics of children with ASD are more pronounced compared to their non‐ASD counterparts. In the line graph of intelligence scale scores (as shown in Figure 2), children with LF‐ASD and HF‐ASD exhibit the highest scores in Object Assembly and Block Design, reflecting their visual‐spatial abilities. This finding aligns with previous studies on HF‐ASD [19, 33, 34], where the curve demonstrates significant fluctuations with notable peaks and troughs, whereas the ID and TD groups show a relatively stable pattern. Subsequently, an analysis of the proportions of peak and trough values in the subtest scores of children with ASD across different diagnoses further corroborates earlier findings [18, 21], indicating that HF‐ASD children tend to have peak and valley values predominantly in Block Design and Picture Completion tasks, whereas LF‐ASD children show peak and valley values mainly in Object Assembly and Arithmetic tasks. This may be related to the fact that children with ASD typically exhibit strong abstract, logical thinking, and reasoning abilities, but face challenges in attention to detail, lack of a global perspective (as seen in Picture Completion), non‐verbal associative learning (imitation), and sequential arrangement and storytelling (as seen in Picture Arrangement) [35, 36]. Both HF‐ASD and LF‐ASD children demonstrate a correlation in Block Design, Arithmetic, and Digit Span with social‐related scales (such as SRS and ADOS), indicating that the visual strengths of children with ASD can be effectively utilized to visualize concrete objects for intervention and learning, a core characteristic of ASD [19, 37]. The findings of this study provide the first evidence that individuals with LF‐ASD exhibit visual characteristics similar to those with HF‐ASD [11, 28, 38], which can be leveraged for developing intervention strategies targeted at low‐functioning individuals.

In clinical practice, particularly children diagnosed with LF‐ASD, often conceals their strengths due to associated intellectual disabilities, which can lead to feelings of inferiority among children and anxiety of their parents. Consequently, this study employs the “peaks and valleys” characteristic of the WISC, in conjunction with clinical histories, to aid physicians and parents in recognizing that children with ASD possess both cognitive strengths and weaknesses. This understanding can transform intervention strategies for ASD, alleviating parental concerns and providing a reference for the clinical diagnosis and differentiation of ASD children. Moreover, in the intervention of children with ASD, guiding parents and teachers to fully harness children's strengths can facilitate the addressing of weaknesses, thereby enhancing communication during the cultivation of children's abilities. Ultimately, this approach may improve children's social functioning and language expression skills, potentially influencing the future career planning of children with ASD and offering new directions for intervention.

## 5 Limitations

This study has several limitations. Firstly, the scale employed in our analysis, C‐WISC, encompasses fewer dimensions for assessing intelligence compared to the Fourth or Fifth Edition of the Wechsler Intelligence Scale for Children (WISC‐IV/V). In future research, we will validate the findings of this study using the latest assessment scales. Furthermore, as a single‐center retrospective study, this research is subject to potential regional differences and selection bias due to sample size imbalance and lack of data on socioeconomic status and educational background. To enhance the reliability of our data, we will collect cases from additional hospitals or regions, and supplement the missing demographic characteristics of the sample. Last, the clinical scale utilized in this study is a behavioral observation scale, which may introduce measurement errors. Future research should utilize objective diagnostic or screening tools to reduce recall and reporting biases introduced by self‐report scales, such as eye‐tracking technology, near‐infrared brain imaging, and functional magnetic resonance imaging, to explore the relationship between the intellectual structure characteristics of children with ASD and brain development, attention, language ability, etc.

## 6 Conclusion

This study analyzes the intellectual structural characteristics of children with ASD at different levels of intelligence, indicating that both LF‐ASD and HF‐ASD children exhibit significant imbalances, characterized by a peak‐to‐valley difference of at least 2 SD. The “peaks” are predominantly reflected in visual advantage tasks such as Block Design and Object Assembly, whereas the “valleys” are primarily evident in tasks such as Picture Completion and Arithmetic. Moreover, LF‐ASD children display more severe core symptoms compared to HF‐ASD. This finding offers new insights for the clinical differentiation and individualized intervention strategies for children with ASD.

## Author Contributions

**Danyang Zhang:** methodology, conceptualization, formal analysis, investigation, resources, writing – original draft, visualization. **Qiuhong Wei:** methodology, writing – review and editing. **Binyue Hu:** resources. **Dan Ai:** resources. **Yu Zhang:** resources. **Xueli Xiang:** resources. **Ting Yang:** resources, supervision, project administration. **Qian Zhang:** resources. **Qian Chen:** resources. **Min Guo:** resources. **Jie Chen:** supervision, project administration, writing – review and editing. **Tingyu Li:** conceptualization, resources, funding acquisition, supervision, project administration, writing – review and editing. **Hua Wei:** resources, writing – review and editing, supervision.

## Funding

This work was supported financially by the National Natural Science Foundation of China (No.81771223), and Chief Medical Expert Studio of Chongqing (No.YWBF[2018]263).

## Ethics Statement

This study has been approved by the Ethics Committee of the Children's Hospital Affiliated with Chongqing Medical University (2024305), and the requirement for informed consent has been waived.

## Conflicts of Interest

The authors declare no conflicts of interest.

## Data Availability

Public release of the raw data at this early stage could compromise these ongoing academic efforts so the data are not shared so far.

## Appendix A

**TABLE A1:** Analysis of sex distribution in the HF‐ASD and LF‐ASD groups.

Variables		Diagnostic, *n* (%)		Total	*χ* ^2^	*p*
		HF‐ASD	LF‐ASD			
Gender	Male	100 (81.97)	87 (83.65)	187 (82.74)	0.112	0.738
	Female	22 (18.03)	17 (16.35)	39 (17.26)		
Total		122	104	226		

Abbreviations: HF‐ASD, high‐functioning autism spectrum disorder; LF‐ASD, low‐functioning autism spectrum disorder.

**TABLE A2:** Analysis of age distribution in the HF‐ASD, LF‐ASD, TD and ID groups (Dunn's *t*‐test).

Variable	Diagnostic (I)	Median (I)	Diagnostic (J)	Median (J)	Difference (I‐J)	*p*
Age	HF‐ASD	8.410	LF‐ASD	8.340	0.070	0.506
	HF‐ASD	8.410	ID	8.410	0.000	0.831
	HF‐ASD	8.410	ID	7.420	0.990	0.278
	LF‐ASD	8.410	ID	8.340	0.070	0.475
	LF‐ASD	8.340	TD	7.420	0.920	0.850
	ID	8.410	ID	7.420	0.990	0.382

Abbreviations: HF‐ASD, high‐functioning autism spectrum disorder; ID, intellectual disabilities; LF‐ASD, low‐functioning autism spectrum disorder; TD, typical development.

**TABLE A3:** Analysis of paired *t*‐tests for VIQ and PIQ in the HF‐ASD and LF‐ASD groups.

Name	Paired groups (mean ± SD)		Difference (VIQ–PIQ)	*t*	*p*
	VIQ	PIQ			
HF‐ASD	90.93 ± 18.49	97.75 ± 13.16	−6.83	−4.647	< 0.001
LF‐ASD	57.13 ± 8.27	65.04 ± 9.60	−7.91	−7.397	< 0.001

Abbreviations: HF‐ASD, high‐functioning autism spectrum disorder; LF‐ASD, low‐functioning autism spectrum disorder; PIQ, performance intelligence quotient; VIQ, verbal intelligence quotient.

**TABLE A4:** Differences between HF‐ASD and LF‐ASD groups in different subtests in C‐WISC.

Variables	Diagnostic median (P25, P75)		*U*	*z*	*p*
	HF‐ASD (*n* = 122)	LF‐ASD (*n* = 104)			
Information	7.000 (6.0, 10.0)	4.000 (3.0, 5.0)	1544.000	−9.873	0.000
Similarities	10.000 (7.0, 13.0)	3.500 (2.0, 7.0)	1521.500	−9.870	0.000
Arithmetic	9.000 (5.0, 11.0)	1.000 (0.0, 2.8)	704.500	−11.602	0.000
Vocabulary	7.000 (6.0, 10.0)	6.000 (5.0, 6.0)	2933.000	−7.058	0.000
Digit span	9.000 (7.0, 11.0)	1.000 (0.0, 5.0)	1043.500	−10.880	0.000
Picture completion	7.000 (6.0, 8.0)	5.000 (4.0, 5.0)	1921.500	−9.148	0.000
Picture arrangement	8.000 (7.0, 10.0)	6.000 (5.0, 6.0)	1465.000	−10.052	0.000
Block design	12.000 (9.0, 15.0)	6.000 (4.0, 6.0)	928.000	−11.103	0.000
Object assembly	12.000 (8.8, 14.0)	7.000 (5.0, 8.0)	1835.000	−9.243	0.000
Coding	9.000 (7.0, 11.0)	4.000 (2.0, 7.0)	1508.500	−9.904	0.000

Abbreviations: HF‐ASD, high‐functioning autism spectrum disorder; LF‐ASD, low‐functioning autism spectrum disorder.

**TABLE A5:** Quantitative analysis of the differential scores of the C‐WISC scale across different diagnoses.

C‐WISC scale	Group	Mean	Relationship with multiples of the standard deviation (SD)	*F*	*p*
Difference	HF‐ASD	9.27	3.09 SD	71.428	0.000
	LF‐ASD	7.95	2.65 SD		
	ID	5.47	1.82 SD		
	TD	5.73	1.91 SD		

Abbreviations: HF‐ASD, high‐functioning autism spectrum disorder; ID, intellectual disabilities; LF‐ASD, low‐functioning autism spectrum disorder; TD, typical development.

**TABLE A6:** Analysis of the correlation between the scores of various subtests of the C‐WISC and the auxiliary diagnostic scales (S‐M, SRS, ADOS) (*r*).

Scales diagnostic	Variables	Information	Similarities	Arithmetic	Vocabulary	Digit span	Picture completion	Picture arrangement	Block design	Object assembly	Coding
S‐M	HF‐ASD	0.300**	0.229*	0.215*	0.235*	0.290**	0.151	0.124	0.230*	0.178	0.085
	LF‐ASD	0.080	0.295**	0.324**	0.140	0.318**	0.084	0.089	0.003	0.301**	0.320**
SRS	HF‐ASD	−0.241*	−0.180	−0.189	−0.113	−0.197	−0.217*	−0.197	−0.231*	−0.065	−0.070
	LF‐ASD	−0.113	−0.472**	−0.226	−0.153	−0.436**	−0.093	−0.192	−0.238*	−0.084	−0.431**
ADOS total score	HF‐ASD	−0.402**	−0.451**	−0.450**	−0.339*	−0.408**	−0.368*	−0.152	−0.586**	−0.533**	−0.020
	LF‐ASD	−0.104	−0.359**	−0.446**	0.267*	−0.547**	−0.179	−0.203	−0.451**	−0.175	−0.445*

*Note:* The values in the table all represent correlation coefficients value (*r*).

Abbreviations: ADOS, autism diagnostic observation schedule; HF‐ASD, high‐functioning autism spectrum disorder; ID, intellectual disabilities; LF‐ASD, low‐functioning autism spectrum disorder; S‐M, infant‐junior high school student social life ability scale; SRS, social responsiveness scale; TD, typical development.

**p* < 0.05.

***p* < 0.01.
